# Pancreatic human islets and insulin-producing cells derived from embryonic stem cells are rapidly identified by a newly developed Dithizone

**DOI:** 10.1038/s41598-019-45678-y

**Published:** 2019-06-26

**Authors:** Bashar Khiatah, Meirigeng Qi, Youjun Wu, Kuan-Tsen Chen, Rachel Perez, Luis Valiente, Keiko Omori, Jeffrey S. Isenberg, Fouad Kandeel, Jiing-Kuan Yee, Ismail H. Al-Abdullah

**Affiliations:** Department of Translational Research and Cellular Therapeutics, Diabetes and Metabolism Research Institute, Beckman Research Institute, City of Hope, Duarte, USA

**Keywords:** Stem-cell research, Embryonic stem cells

## Abstract

We developed an optimized Dipheylthiocarbazone or Dithizone (DTZ) with improved physical and chemical properties to characterize human islets and insulin-producing cells differentiated from embryonic stem cells. Application of the newly formulated iDTZ (i stands for islet) over a range of temperatures, time intervals and cell and tissue types found it to be robust for identifying these cells. Through high transition zinc binding, the iDTZ compound concentrated in insulin-producing cells and proved effective at delineating zinc levels *in vitro*.

## Introduction

The pancreas is composed of endocrine islets and exocrine acinar and ductal cells. The endocrine islets synthesize and secrete insulin in response to secretagogues to control and regulate glucose homeostasis. Islets consist of α, β, δ, PPP, and € cells that secret glucagon, insulin, somatostatin, pancreatic polypeptide and ghrelin respectively. The exocrine acinar cells secrete proteases, lipase and amylase for digestion of proteins, fats and carbohydrates^1–4^.

Individuals with type 1 diabetes (T1D) experience auto-immune-mediated destruction of insulin-producing β-cells and consequently require life-long insulin therapy^5^. Islet transplantation (IT) is proven to be an effective treatment for individuals with brittle T1D^6^. However, the success of IT is reliant on the quality and number of islets^7^. Thus, rigorous methods for pre-transplant islet characterization are needed. One such technique employs DTZ, that stains high-zinc containing β-cells red in contrast to unstained acinar and ductal cells, and is a standard approach for islet charcterization^8^. However, current DTZ formulations are highly unstable, must be prepared fresh daily and have essentially no shelf-life. We evaluated a new DTZ formulation (iDTZ for islet DTZ) that showed improved solubility, optimized staining features and enhanced reproducibility and that permitted automated analysis of human islet quality.

**Figure 1:**
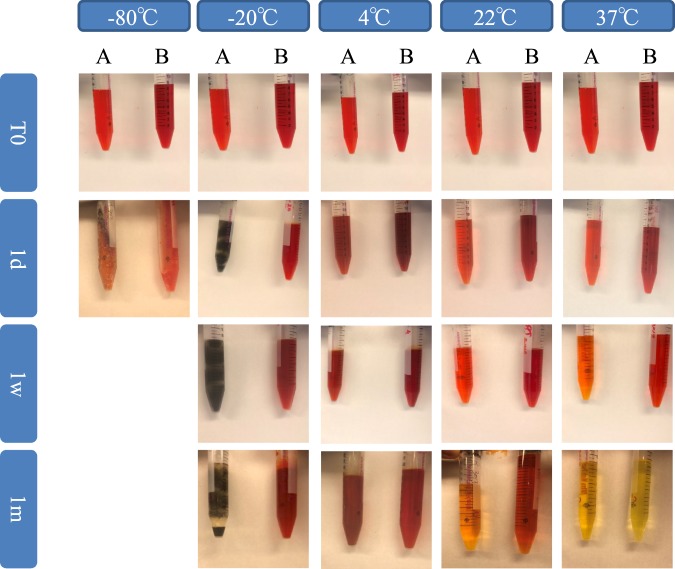
Solutions of iDTZ show improved stability under various temperatures and lengths of time. Solutions were prepared as described using standard DTZ (**A**) and iDTZ (**B**) and exposed to a range of temperatures and storage intervals (−80°, −20°, 4 °C, and 22 °C and 1, 7, and 30 days).

Currently, DTZ solutions require same-day preparation due to the tendency of the reagent to precipitate once in solution. The instability of these solutions makes it difficult to characterize islet β-cells^9^ as well as other cell types like yeast^10^. In contrast to existing DTZ formulations, iDTZ was found to be stable and highly soluble in solution. Solutions of iDTZ tolerated storage at −20 to 22 °C with no change in solution color or precipitation for up to one month post-preparation (Fig. 1). Further, solutions of iDTZ stored at 37 °C were stable while standard formulation DTZ solutions degraded within one week of preparation (Fig. 1). Thus, solutions made with iDTZ have improved physical and chemical stability within a temperature range encountered during normal usage and handling.

Zinc is an important element present within the insulin granules of the β-cells and has been shown to be involved in diabetes, cancer, neurodegeneration and wound healing^11^. Zinc inhibits cell apoptosis, and serum- and tissue-free culture media contain zinc as a supplement to improve cell survival and function. Islets contain zinc ions (Zn^2+^) and the zinc transporter channels ZnT and ZIP^12^ are abundant in β-cells^13^. Zinc ions are essential in maintaining the structure and integrity of insulin molecules within β-cells and are secreted with insulin^14–16^. DTZ is relatively insoluble in water but solubilizes partially in ethanol, chloroform and DMSO. DTZ readily complexes with transition metals, including zinc, and can identify zinc-replete cells such as islets^17^, and can distinguish islets from exocrine and ductal cells^18^. For these reasons, DTZ staining is a standard technique for determining the number of islets in pancreatic digest and for assessing islet quality^18^. Traditionally, this process involved manual assessment of DTZ-stained islets and was inherently variable. At the same time, studies employing dithizone analogues are few. One such DTZ analogue, 1,5-Bis(2,5-difluorophenyl)-3-mercaptoformazan, was noted to have greater affinity for Co^2+^ and Hg^2+^ compared to Zn^2+^ and has not been used to identify islets^19^. Interestingly, the analogue [11C]-DTBZ, while not used to stain isolated islets, was used to image islets *in vivo*^20^.

**Figure 2:**
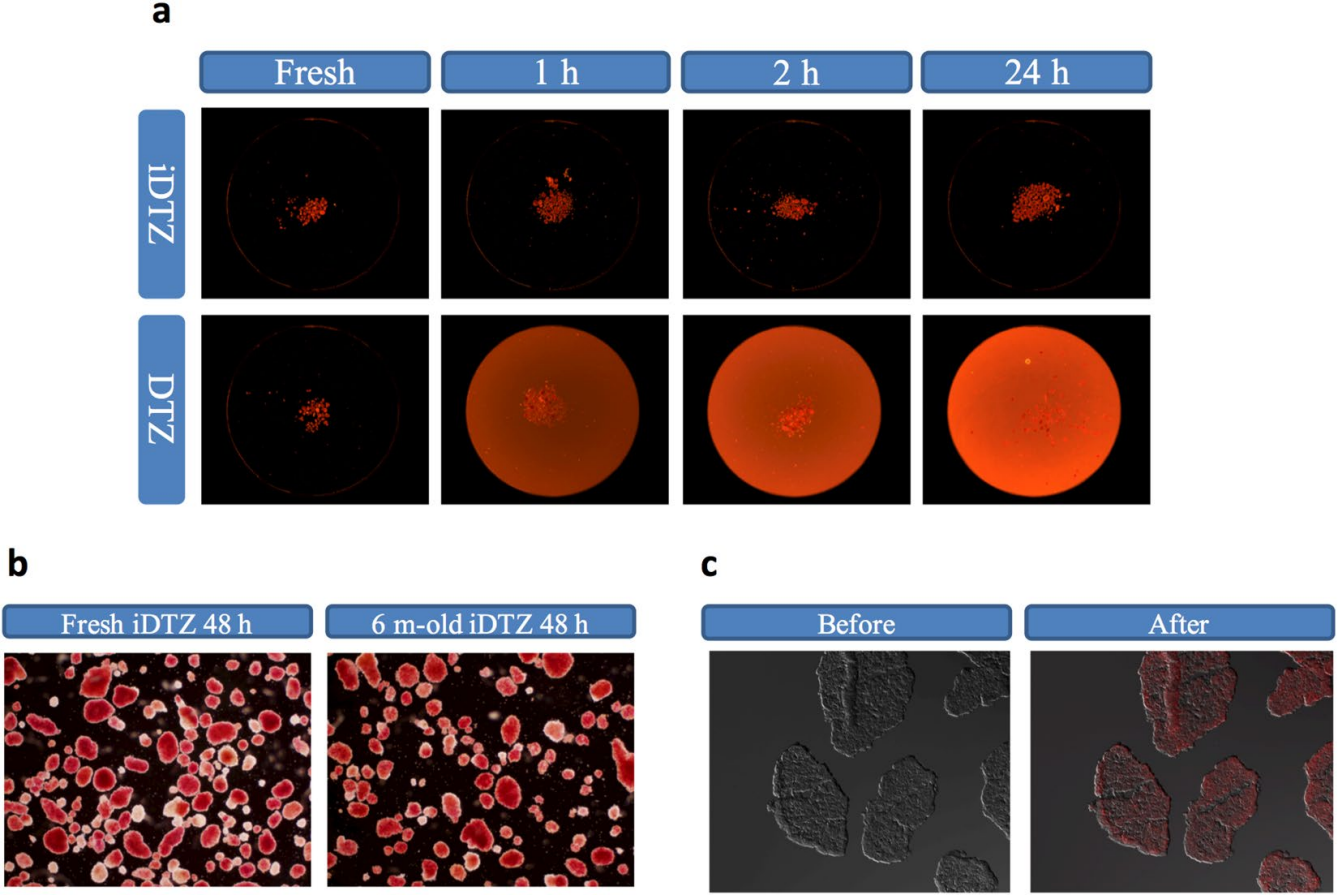
Solutions of iDTZ show improved islet staining under various conditions employing an automated quantification system. Human islets stained with iDTZ or standard DTZ solution visualized at the indicate time points (**a**). Human islets stained with fresh iDTZ solution and islets stained with iDTZ solution stored for 6 months at −20 °C (**b**) and visualized 48 hours post-staining. Human islet cryo-sections were stained with iDTZ solution (**c**). Representative pictures from three experiments are shown. All the pictures were obtained using an Olympus microscope.

Automated methods for the quantification of stained islets have been reported with mixed results^9,21–23^. We hypothesized this was secondary to limitations in standard DTZ solution. We tested this hypothesis comparing standard DTZ to iDTZ solution under varied conditions and parameters^9^. Interestingly, over 24 hours, islets stained with iDTZ displayed normal morphology and minimal increases in background staining. In contrast, islets stained with standard DTZ displayed intense background staining preventing automated quantification (Fig. 2a). In this regard, iDTZ solution is distinct in supporting islet quality validation over an extended period of time, an important factor in qualifying islets for clinical transplantation. Six months after preparation, solutions of iDTZ were found to provide islet-specific staining on par with freshly made solutions, an effect that persisted for 48 hours (Fig. 2b). These findings indicate that iDTZ staining creates a stable color signal that will allow for greater sharing of stained biomaterial without signal degradation.

Both the pancreas and prostate have abundant concentrations of zinc compared to other organs^24^, whereas prostate cancer is associated with decreased zinc levels^25^. However, we found that solutions of iDTZ were sufficient for localizing islet-based zinc in pancreatic cryo-sections (Fig. 2c). These findings suggest that this reagent could be used as a simple, rapid means for detecting zinc in tissues. Further, the process of isolating islets from cadaveric pancreata is complex, expensive and lengthy. Given this, employing iDTZ solution to stain pancreatic cryo-sections prior to proceeding with islet isolation would be advantageous, especially when considering islet isolation from pancreata from individuals with high HbA1c, chronic diabetes and extensive insulin usage^26^.

**Figure 3:**
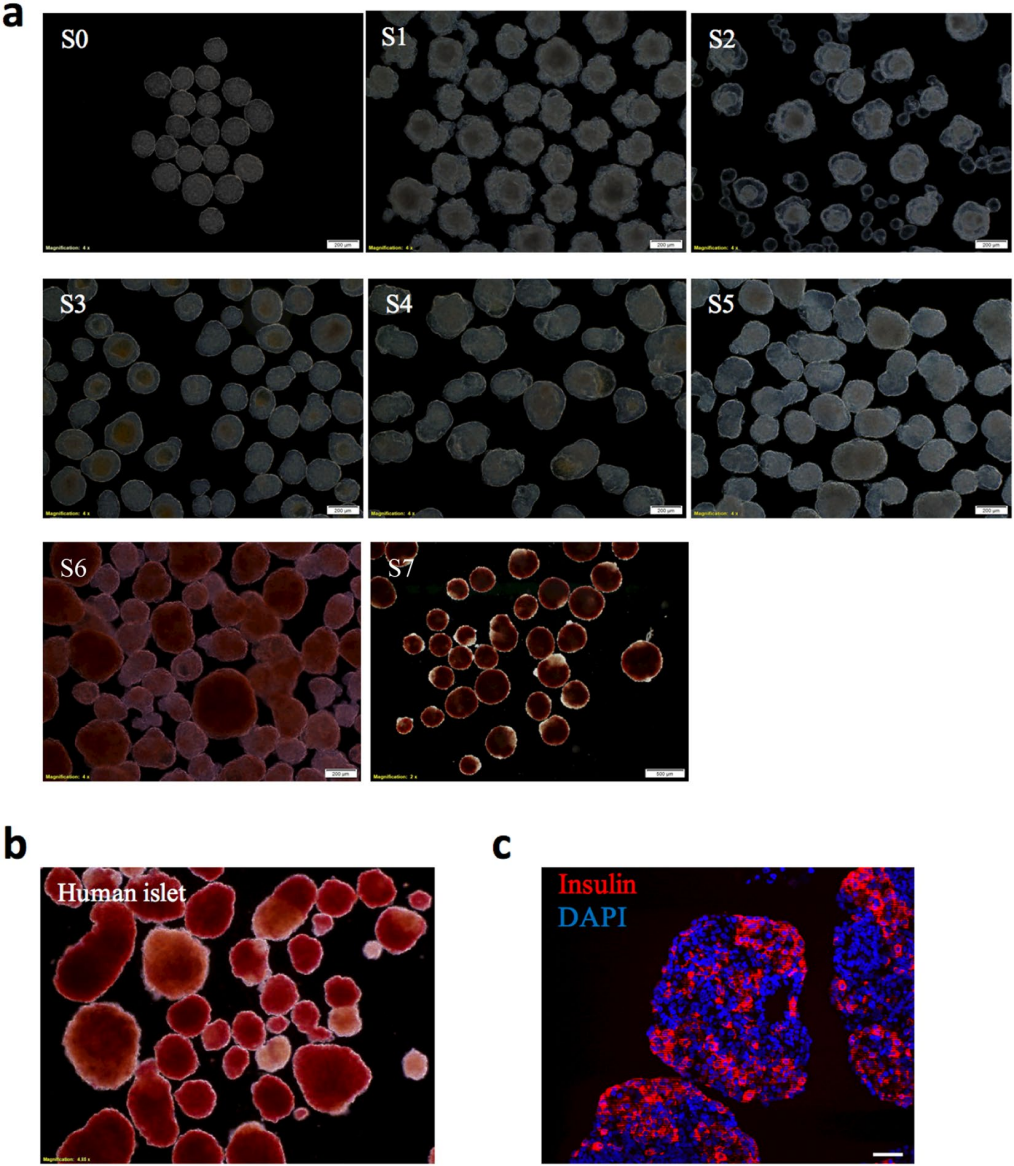
Human islets and insulin-producing cells differentiated from embryonic stem cells are rapidly identified by iDTZ. Stage 6 and 7 cell clusters were stained positively by iDTZ as compared to other stages (1–5) (**a**). Human islets stained with iDTZ (**b**). Immuno-fluorescent staining of stage 7 differentiated human embryonic stem cells showing insulin (red), and DAPI staining of the nucleus (blue), (**c**). The scale bar represents 50 μm.

The application of standard DTZ solutions beyond staining of mature islets is very limited. In one report, standard DTZ was used to stain stem cell-derived insulin-producing cells^27^. This suggested that stem cell-derived islet-like cells, on differentiation, may display increased levels of zinc and insulin. To interrogate this idea, we utilized the human embryonic cell line H1^28^. Cells were cultured under appropriate conditions for differentiation into insulin-producing cells using an established 7-stage protocol^28^. Clusters of cells were collected at every differentiation stage and stained with iDTZ. At stage 7, cells displayed a classic islet phenotype and were positive for intra-cellular zinc (Fig. 3a,b), concurrent with the presence of immuno-reactive insulin (Fig. 3c), a finding previously noted in stage 7 cells^28^. Stage 7 clusters, despite staining with iDTZ, were noted to be de-granulated compared to mature adult human islets, indicating that stage 7 cell clusters would need further steps to attain full maturation and development (Fig. 3). Interestingly, we found that approximately 10% of stage 6 cells retained iDTZ, indicating that a portion of such cells are immature insulin-producing cells. Thus, iDTZ can be employed as a simple and robust method to identify fully mature insulin-producing β-cells prior to transplantation into recipients. Parenthetically, iDTZ could be used as a rapid and simple agent to detect insulin producing cells transduced by several methods^29,30^.

**Figure 4:**
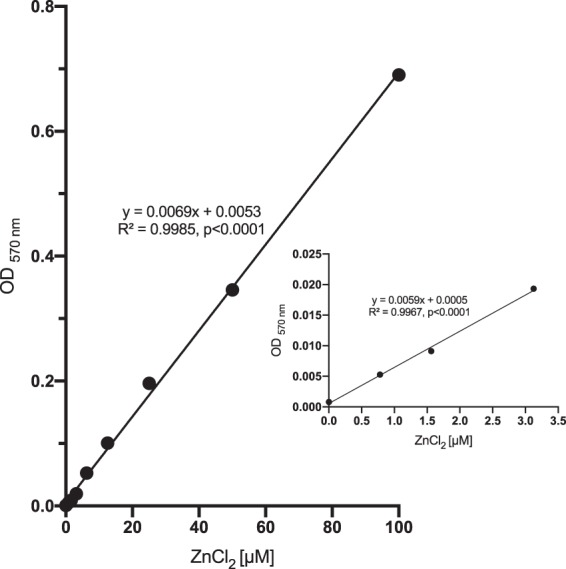
Zinc levels can be measured using iDTZ. There was a highly significant correlation between zinc concentration and the absorbance readings at 570 nm (R^2^ = 0.9985, p < 0.0001).

Currently, characterization of zinc in liquids is done using a double beam flame absorption spectrophotometer or commercially available kits. However, these methods are complex and expensive. To further characterize the sensitivity of iDTZ to measure zinc levels we developed a standard curve and measured dye levels at 570 nm in samples containing known concentrations of zinc chloride (Fig. 4). As these data indicate, iDTZ can be used in microplate and plate readers available in most laboratories. Extrapolating these results, it is likely that solutions of iDTZ could detect zinc in a range of tissue and fluid samples. Additionally, solutions of iDTZ may find application in detecting other metals such as lead, mercury, and cadmium.

## Methods

### Isolation of human pancreatic islets

Human islets were isolated from cadaveric donor pancreata obtained from a local organ procurement organization following a standard operational procedure^31^. The use of human islets was approved by the Institutional Review Board of City of Hope and informed consent was obtained from the legal next of kin of each donor. All islets were cultured for 24–48 hrs at 37 °C, 5% CO_2_ prior to staining with iDTZ or standard DTZ. All experiments were performed in accordance with relevant guidelines and regulations.

### Preparation of DTZ and iDTZ

Standard DTZ solutions were prepared by dissolving 80 mg of Dithizone (Sigma, Cat #D5130 – 50 G) in 10 mL of DMSO (Fisher Chemical, D128-1) and vortexing for 30 seconds. Solutions were then left at room temperature (22 °C) for 10 minutes. Using a 0.2 µm filter, 40 mL of DPBS (Ca^2+^ and Mg^2+^ free) was then added to the solution. To remove any visible precipitate, the final DTZ solution was passed numerous times through a 0.22 μm filter. iDTZ was prepared by dissolving 80 mg of DTZ (Sigma, Cat #D5130) in a solution containing 62% DMSO, 37.5% methanol (Fisher, Cat #UHPLC-MS), and 0.5% ammonium hydroxide solution (Sigma, Cat #338818). iDTZ solution did not require filtration and is available commercially (Gemini, Cat #900-755). The iDTZ solution can be used to stain islets and clusters of cells derived from ES at a 1:10 or greater dilution.

### Evaluation of standard DTZ and iDTZ solution stability

To compare the stability of both solutions in response to variations in temperature and time, solutions of standard DTZ and iDTZ were exposed to a range of temperatures: −80 °C, −20 °C, 4 °C, 22 °C, and 37 °C. Visual analysis of solutions at each temperature was then undertaken at day 0, 7 and 30. Pictures were taken for each condition.

### Assessment of cell staining by standard DTZ and iDTZ solutions

To evaluate the staining efficacy, solutions were prepared fresh and used for staining isolated islets. Islet morphology was observed and imaged using an automated islet cell counter (ICC) (Biorep Technologies, Miami, USA). Briefly, 100 μL of islet sample was pipetted (Drummond pipette, Fisher Scientific, USA) into an ICC-provided dish^9^. A 250 μL aliquot of standard DTZ or iDTZ solution was then added and the dish agitated gently prior to placement on the imaging stage with the temperature at 22 °C. Data was acquired at time zero (T0), 1 hr (T1), 2hrs (T2) and 24hrs (T24).

### Assessment of islet staining with stored iDTZ solution

Human islets were stained with iDTZ solution that was stored for 6 months at −20 °C prior to use. In control experiments, freshly made iDTZ solution was used to stain islets from the same cell preparation.

### Assessment of zinc and insulin status in differentiated stage 7 human embryonic stem cells

Human embryonic stem cells were differentiated following a previously published method^28^. Briefly, human ES cell line H1 cells (WiCell Research Institute, Inc. Madison, WI, USA) were cultured at 37 °C, 5% CO_2_ on Matrigel-coated plates (BD BioScience, Cat #354671) in mTeSR™1 Complete Kit medium (StemCell Technologies, Vancouver, Canada, Cat #85850) that was changed daily until cells were confluent. Cultured H1 cells were dissociated into single cells by incubation with Accutase (STEMCELL Technologies, Vancouver, Ca., Cat #07920) for 5–10 minutes at 37 °C. Single cells were counted and 5.5 × 10^6^ cells in 5.5 mL mTeSR medium supplemented with 10 µM Y27632 (Stemgent, 04-0012-H-10) were seeded into 6-well low-attachment plates (Costar, Cat #3471), placed on an orbital shaker (New Brunswick Innova 2000, #M1190-0000) set to 100 rpm to promote cluster formation and then cultured at 37 °C, 5% CO_2_. Forty-eight hours later, the culture medium was changed into differentiation medium. Stage-specific differentiation was conducted by the controlled addition of specific small molecules as described^28^. At each stage, representative clusters were transferred into new 6-well culture dishes and washed 3 times with 3 mL of COHSII (Gemini, Sacramento, CA, Cat#900-7500) prior to staining with iDTZ. After 3 minutes, the cells were washed 3 times with 3 mL of COHSII and suspended with COHSII. Cell clusters were examined using a ckx31 Olympus microscope and pictures were acquired using a camera. The iDTZ solution employed was a single batch stored at −20 °C until use. Human islets stained with iDTZ were used as control.

### Measurement of zinc concentration using iDTZ

ZnCl_2_ (Sigma, CAS: 7646-85-7) was dissolved using MQH_2_0 to prepare a concentration of 200 µM. To test the sensitivity of the iDTZ, 50 μL aliquots of iDTZ [500 μM] were pipetted into wells of a black flat-bottom 96-well plate (Corning, Cat #3631) along with a 50 μL aliquot of nine concentrations of ZnCl_2_ (final concentration = 100, 50, 25, 12.5, 6.25, 3.125, 1.56, 0.78, 0 μM). Control wells contained 50 µL of 500 µM of iDTZ and 50 µL of water served as the blank. Each concentration was replicated twelve times. After a 15 minute incubation at room temperature, the plate was read at 570 nm using a Tecan infinite M200 (Tecan Group Ltd, Männedorf, Switzerland) plate reader and a linear standard curve was established.
